# Immunomodulation in the canine endometrium by uteropathogenic *Escherichia coli*

**DOI:** 10.1186/s13567-016-0396-z

**Published:** 2016-11-09

**Authors:** Sofia Henriques, Elisabete Silva, Marta F. Silva, Sandra Carvalho, Patrícia Diniz, Luís Lopes-da-Costa, Luisa Mateus

**Affiliations:** 1 Reproduction and Development Laboratory, CIISA, Faculty of Veterinary Medicine, University of Lisbon, Lisbon, Portugal; 2 Pathology Laboratory, CIISA, Faculty of Veterinary Medicine, University of Lisbon, Lisbon, Portugal

## Abstract

This study was designed to evaluate the role of *E. coli* α-hemolysin (HlyA) in the pathogenesis of canine pyometra, and on the immune response of canine endometrial epithelial and stromal cells. In Experiment 1, the clinical, hematological, biochemical and uterine histological characteristics of β-hemolytic and non-hemolytic *E. coli* pyometra bitches were compared. More (*p* < 0.05) metritis cases were observed in β-hemolytic *E. coli* pyometra uteri than in non-hemolytic *E. coli* pyometra uteri. β-hemolytic *E. coli* pyometra endometria had higher gene transcription of *IL*-*1β* and *IL*-*8* and lower gene transcription of *IL*-*6* than non-hemolytic *E. coli* pyometra endometria (*p* < 0.01). In Experiment 2, the immune response of endometrial epithelial and stromal cells, to hemolytic (Pyo18) and non-hemolytic *E. coli* strains (Pyo18 with deleted *hlya*-Pyo18Δ*hlyA*- and Pyo14) were compared. Following 4 h of incubation, Pyo18 decreased epithelial cell numbers to 54% (*p* < 0.001), and induced death of all stromal cells (*p* < 0.0001), whereas Pyo18Δ*hlyA* and Pyo14 had no effect on cell numbers. Compared to Pyo18*ΔhlyA* and Pyo14, respectively, Pyo18 induced a lower transcription level of *IL*-*1β* (0.99 vs 152.0 vs 50.9 fold increase, *p* < 0.001), *TNFα* (3.2 vs 49.9 vs 12.9 fold increase, *p* < 0.05) and *IL*-*10* (0.4 vs 3.6 vs 2.6 fold increase, *p* < 0.001) in stromal cells, after 1 h of incubation. This may be seen as an attempt of hemolytic *E. coli* to delay the activation of the immune response. In conclusion, endometrial epithelial and stromal cell damage induced by HlyA is a potential relevant step of *E. coli* virulence in the pathogenesis of pyometra.

## Introduction

Pyometra is a common diestrous disease of bitches. *Escherichia coli* is isolated from the uterus of up to 90% of bitches with pyometra [1, 2] and its presence is associated with severe systemic signs and a potentially life-threatening condition. The systemic inflammatory response syndrome (SIRS) is detected in more than 50% of bitches with *E. coli* pyometra [3]. These *E. coli* isolates derive from the host’s fecal and perineal flora [4], being mainly assigned to phylogenetic group B2, and characterized by a high number of uropathogenic *E. coli* (UPEC) virulence factor (VF) genes and pathogenicity-associated islands markers [2]. The α-hemolysin (*hlyA*) VF gene was detected in 35–52% of *E. coli* pyometra cases [2, 5, 6]. Although this prevalence leads to the suggestion that α-hemolysin (HlyA) contributes to the virulence of *E. coli* strains, the role of this toxin in the pathogenesis of canine pyometra is unknown. HlyA is a RTX pore-forming exotoxin. At high concentrations, HlyA is able to lyse erythrocytes and nucleated host cells. At sublytic concentrations, HlyA can disrupt the immune signaling and cytoskeletal components [7].

Toll-Like-Receptor (TLR)-mediated immune surveillance is an important component of the defence mechanisms within the canine uterus [8]. Up-regulation of TLR2 and four transcription [9, 10] and expression [8] was observed in *E. coli* pyometra endometrium. Uterine inflammatory response towards *E. coli* is associated with an endometrial up-regulation of genes related with chemokines, cytokines, inflammatory cell extravasation, anti-bacterial action, proteases and innate immune response [9, 11]. In accordance, pyometra is characterized by endometrial tissue damage, infiltration by inflammatory cells, accumulation of pus, and increased expression of inflammatory mediators such as interleukins *IL*-*1β*, *IL*-*6* and *IL*-*8* [9, 11]. Different expression patterns of cytokines were observed in bitches with or without pyometra-associated SIRS [3].

Most of the studies on canine pyometra were carried out at the time of diagnosis, a late stage of the disease, and did not take into account the virulence background of *E. coli*. The characterization of the endometrial cell cytokine response to *E. coli* may lead to a relevant insight into the pathogenesis of pyometra. Additionally, the characterization of the role of specific *E. coli* VF genes in the modulation of the endometrial immune response and on the pathogenicity of the bacterium may prove rewarding in the development of novel diagnostic and therapeutic approaches to the disease. In this regard, HlyA becomes a promising candidate.

This study includes two experiments. Experiment 1 was designed to evaluate and compare the clinical and uterine histological and immune response gene transcription of hemolytic and non-hemolytic *E. coli* pyometra bitches. Prompted by results of Experiment 1, Experiment 2 was designed to evaluate the effects of *E. coli* HlyA on the modulation of the inflammatory response of canine endometrial epithelial and stromal cells.

## Materials and methods

### Experiment 1

Healthy diestrous (*n* = 10) and pyometra (*n* = 18) bitches presented to the Hospital of the Faculty of Veterinary Medicine of the University of Lisbon were selected for this experiment. The average age of bitches was 8 years (range 3–13 years). Healthy bitches were submitted to elective ovariohysterectomy (OHE) for contraceptive purposes, as requested by owners. In pyometra bitches the diagnosis was based on case history, clinical signs and the ultrasonographic finding of an enlarged, fluid-filled uterus. A blood sample for hematologic (Hemogram) and biochemical (urea, creatinine, phosphatase alkaline, alanine-aminotransferase) analysis was collected prior to OHE. After removing the uterus, an intra-uterine swab was processed for bacteriological analysis. Selection of the 18 pyometra bitches was based on the isolation of hemolytic (*n* = 8) and non-hemolitic (*n* = 10) *E. coli* strains. Uterine samples were collected as described previously [8] and either fixed for 24 h in 4% neutral phosphate buffered formalin (for IHC, described in sub-section “Immunohistochemistry”) or immersed for 24 h in RNAlater (Qiagen, GmbH, Hilden, Germany) and then stored at −80 °C (for RT-PCR and qRT-PCR, described in sub-section “RNA extraction, cDNA synthesis, RT-PCR and qRT-PCR”).

#### Immunohistochemistry

Immunohistochemistry (IHC) was used to identify T lymphocytes (Rabbit polyclonal anti-human CD3, diluted 1:200; Dako, Glostrup, Denmark), B lymphocytes (mouse monoclonal anti-human anti-CD79 αcy, clone HM57, diluted 1:150; Dako) and granulocytes and macrophages (mouse anti-human MCA874G, clone MAC387, diluted 1:400, Dako) in uterine samples. Except for anti-CD3 antibody, all protocol steps were carried out using the Novolink Polymer Detection System (Novocastra, Leica Biosystems, Newcastle, UK), according to the manufacturer’s instructions. The antigen retrieval step was performed by microwave treatment (3 × 5 min) in Tris–EDTA buffer (pH 9.0). After endogenous peroxidase blocking and treatment with protein block solution (Protein Block Solution-Kit NovoLink™), sections were incubated 1 h at room temperature with the respective primary antibodies. CD3 immunostaining was carried out as previously described [8] with minor modifications. Briefly, endogenous peroxidase was quenched by incubating the slides in 3% hydrogen peroxide in water for 30 min followed by antigen retrieval in Tris–EDTA buffer (pH 9.0), as described above. Blocking was performed with blocking solution (PBS + 0.1% Tween + 5% goat serum + 2.5% BSA), for 1 h at room temperature followed by incubation with the primary antibody for 2 h. The peroxidase conjugated goat anti-rabbit IgG polyclonal antibody (diluted 1:100, Dako) was used as secondary antibody. For all antibodies, the staining was developed by incubating the slides with the substrate diaminobenzidine (DAB kit, Zytomed Systems, Berlin, Germany). Staining without the primary antibody and staining with the isotype-matched irrelevant monoclonal antibody were used as negative controls. Human tonsil and dog spleen sections were used as positive control tissues.

### Experiment 2

Uteri were obtained from six healthy bitches (1–3 years old) submitted to ovariohysterectomy (OHE) for contraceptive purposes at the Teaching Hospital of the Faculty of Veterinary Medicine of the University of Lisbon. Only uteri from the first half of diestrus (as determined by vaginal cytology, blood progesterone concentrations and histology), without histological evidence of cystic endometrial hyperplasia (CEH) and a negative bacteriological result were allocated to the study. Three uteri were used for the evaluation of bacterial adhesion and internalization, and the remaining three uteri were used for the evaluation of cytokine transcription, IHC, and the number and morphology of cells following stimulation. All these evaluations considered two technical replicates per uteri for each stimulus (negative un-stimulated control (NUC), LPS, Pyo14, Pyo18, Pyo18Δ*hlyA*).

#### Cell culture

Endometrial epithelial and stromal cell populations were isolated as described by Bläuer et al. [12] and Stadler et al. [13] with some modifications. Briefly, small endometrial strips were digested with 1 mg/mL of collagenase A (Roche Diagnostics GmbH, Mannheim, Germany) and 5 μg/mL of DNAse I (Sigma-Aldrich, St. Louis, USA) in RPMI-1640 (GIBCO^®^, Invitrogen Corporation, New York, USA) supplemented with gentamicin (50 μg/mL, Sigma-Aldrich) and anfotericin B (2.5 μg/mL, Sigma-Aldrich). After 60-90 min of incubation with gently shaking at 37 °C, the cell suspension was filtered through a sterile mesh to remove undigested tissue. The suspension was centrifuged at 200 × *g* for 5 min and re-suspended in 10 mL of culture medium (RPMI-1640 supplemented with 10% heat inactivated fetal bovine serum, 50 μg/mL of gentamicin and 2.5 μg/mL de anfotericin B). Epithelial organoids and stromal fibroblasts were separated by differential centrifugation and filtered through a sterile 40 μm pore size filter. Stromal suspension (bottom of the filter) was pelleted by centrifugation (250 × *g*), freed of red blood cells and suspended in 1 mL of culture medium. Further purification of stromal cells was done by gently pipetting the fraction onto 9 mL of culture medium and letting them to sediment by gravity for 30 min.

Organoid suspension was obtained after back washing the filter with culture medium. After 2 steps of sedimentation by gravity for 5 min, epithelial cells suspension was centrifuged (200 × *g*, 5 min) and the pellet was suspended in 3 mL of trypsin 0.25%—EDTA (GIBCO^®^) with 5 μg/mL of DNAse I. After 2–5 min of incubation, organoids were unbundled with a 26G needle and 2 mL syringe in order to get a single-cell suspension. The viability of stromal and epithelial cells, as assessed by Trypan Blue exclusion dye staining before culture, was 80–95%.

Stromal and epithelial cells were seeded in 24-well plates at a density of 1 × 10^5^ cells/mL and 2 × 10^5^ cells/well/mL, respectively, and cultured in 1 mL of culture medium supplemented with Insulin-Transferrin-Selenium (1×) (ITS liquid media supplement, 100×, Sigma-Aldrich). For stromal cells, medium was changed 12 h after plating to allow selective attachment. For epithelial cells, medium was removed after 24 h to discard nonattached cells. Culture media was then changed every 48 h until cells reached 80% confluence. All cultures were incubated at 37 °C, 5% CO_2_ in air, in a humidified incubator. Purity of stromal and epithelial cells, as determined by their morphology after staining with Giemsa (Accustain^®^, Sigma-Aldrich) in glass coverslips, was respectively 95 and 85%.

#### *E. coli* strains

The two *E. coli* strains used in this study, Pyo14 and Pyo18, were previously isolated from the uterus of pyometra bitches and characterized [14]. Although both strains were from phylogenetic group B2, only Pyo18 carries *hlyA* gene and have a hemolytic phenotype in sheep blood agar [14]. The isogenic *hlyA* deletion mutant was generated in *E. coli* Pyo18 strain using the Lambda-Red recombinase system as described by Datsenko and Wanner [15]. Briefly, the chloramphenicol cassette was amplified from a PKD3 plasmid using primers with 40 nucleotide extension homologous to the *hlyA* gene (*hlyA*1: 5′-cagatttcaatttttcattaacaggttaagaggtaattaagtgtaggctggagctgcttc-3′; *hlyA*2: 5′-cagcccagtaagattgctattatttaaattaataaaatgggaattagccatggtcc-3′). The following PCR conditions were used: initial denaturation of 5 min at 95 °C, 35 cycles of 30 s at 94 °C, 30 s at 50 °C, 2 min at 72 °C and a final extension of 5 min at 72 °C. A purified PCR product was used to replace the chromosomal *hlyA* gene in Pyo18 strain using the helper plasmid pKD46 expressing the lambda recombinase. For this purpose, Pyo18 strain was previously transformed with pKD46 plasmid, which after induction with 0.01 M l-arabinose promotes homologous recombination between the PCR product and the chromosomal target gene. The PCR product was electroporated into Pyo18/PKD46 and the chloramphenicol resistance gene was eliminated using the helper plasmid pCP20. Elimination of pCP20 plasmid was accomplished by bacterial growth at 43 °C. The *hlyA* gene deletion was confirmed by PCR analysis (∆*hlyA1*: 5′-ccattagaggttcttgggc-3′; ∆*hlyA2*: 5′-ggaataaaccaggtaaagtc-3′) and DNA sequencing.

Before stimulation experiments, bacteria were plated onto Columbia agar medium and incubated at 37 °C overnight. For each bacterium, one bacterial colony was inoculated into liquid LB medium at 37 °C for 48 h without agitation, washed three times by centrifugation (5000 × *g* for 5 min) and re-suspended in sterile PBS. The bacterial suspension was diluted at a final working concentration equivalent to 10^8^ CFU/mL. Similar bacterial growth curves were obtained for the three *E. coli* strains (two replicates per strain).

#### *E. coli* cell stimulation

Cell culture conditions were maintained during stimulation. After reaching 80% of confluence, cells were pre-incubated in medium without antibiotic (RPMI-1640 supplemented with 10% of FBS) during 24 h before bacteria inoculation. Endometrial epithelial and stromal cells were incubated with the *E. coli* isolates [multiplicity of infection (MOI) of 6–8], LPS (1 μg/mL *E. coli* O55:B5, Sigma-Aldrich) or medium alone for 1 and 4 h at 37 °C (adhesion step). Cells were washed with PBS to remove non-adherent *E. coli* and further incubated for 1.5 h with fresh medium containing 50 μg/mL gentamicin to eliminate extracellular bacteria (internalization step). The medium was then removed and the cells washed three times with PBS. Cells of two duplicate wells were lysed with 350 µL of RLT/b-ME buffer (Rneasy mini kit, Qiagen GmbH) and frozen at −80 °C until RNA extraction. In parallel plates, after internalization, cells were washed and cell morphology was evaluated. Cells in each well were then trypsinized and the number of viable cells was counted in a Neubauer chamber, using a contrast phase equipped microscope. Results of cell counts for each type of stimulation are presented as the mean percentage relative to the respective unstimulated control.

Adherence and internalization levels were evaluated as described by Letourneau et al. [16]. The percentage of adhered bacteria was calculated by dividing the number of cfu of adhered bacteria by the addition of the number of cfu of adhered and non-adhered bacteria. The percentage of internalization was calculated by dividing the number of cfu of internalized cells by the number of cfu of adhered cells.

#### RNA extraction, cDNA synthesis, RT-PCR and qRT-PCR

Total RNA was isolated from pyometra endometrial tissue and from cell cultures using the Rneasy mini kit (Qiagen GmbH) and DNA digestion was performed with the RNase-free DNase set (Qiagen GmbH). RNA concentration and purity was determined in a NanoDrop 2000/2000c spectrophotometer (Thermo Fisher Scientific, Walthman, USA). Single-stranded complementary DNA (cDNA) synthesis was performed by reverse transcription of 500 ng (endometrial tissue) or 400 ng (cell cultures) of total RNA using the SuperScript III First-Strand synthesis SuperMix for qRT-PCR (Invitrogen, Austin, TX, USA), according to the manufacturers’ protocols.

RT-PCR and qRT-PCR was performed as described by Silva et al. [8]. In Experiment 1, RT-PCR was performed to screen the presence of transcripts of TLR pathway components in endometrial tissue. qRT-PCR was performed to quantify the mRNA levels of cytokines in endometrial tissue (see “Hemolytic and non-hemolytic *E. coli* pyometra endometria have different transcription levels of genes coding pro-inflammatory cytokines (Experiment 1)” section). In Experiment 2, only qRT-PCR was performed to quantify mRNA expression in endometrial cells. Primers (Tables 1 and 2) were first chosen with Primer3 Software and for qRT-PCR confirmed with Primer Express^®^ Software (Applied Biosystems, Foster City, CA, USA). The mRNA transcription of the Ribosomal protein L27 gene (RPL27) had no significant statistical change (*p* > 0.05) in unstimulated (NUC) and stimulated cells (epithelial and stromal), therefore this gene was considered a suitable housekeeping gene. The overall mean transcription (Ct value) of *RPL27* was 20.69 ± 0.59 (Mean ± SD).

**Table 1:** **Primer sequences used for RT-PCR**

Gene	Accession number	Sequence (5′–3′)	Annealing temperature (°C)	Amplicon size (bp)
*MyD88*	XM_534223	FW**-**ACTATCGGCTGAAGTTGTGTGTGT RV**-**TGGTGTAGTCACAGACAGTGATGAA	58.3	276
*TRAM*	NM_001204337	FW-GGGTGTCAGGAAGTCGAAAATA RV-CTGCGTGCAGTATCACAAACTT	56.8	266
*TRAF6*	XM_003432322	FW-AAACTGTGAAAACAGCTGTGGA RV-CAGTTCATGCAAGAAACCTGTC	56.1	564
*TRIF*	XM_849573	FW-CTTCCAAAGCCCATAGAGGA RV-AAGCGGTGTCTTCTACAGGAA	55.4	487
*IRF3*	XM_005616307	FW-GGTGCCTACACTCCTGGAAA RV-CTTCATCAGGCACCAAGAGC	56.0	418
*NFkB*	NM_001003344	FW-TGTTTCACTTGGATCCTTTGAC RV-AGATCCCATCCTCACAGTGTTT	55.4	327
*IL*-*1β*	NM_001037971	FW-CACCAGTGAAATGATGGCTTAC RV-CTCATGTGGAACACCACTTGTT	56.0	453
*IL*-*6*	NM_001003301	FW-GTACATCCTCGGCAAAATCTCT RV-GGATGAGGTGAATTGTTGTGTG	56.0	410
*IL*-*8*	NM_001003200	FW-TTGGCAGCTTTTGTCCTT RV-GGGCCACTGTCAATCACT	52.5	149
*IL*-*10*	NM_001003077	FW-AAGCTGGACAACATACTGCTGA RV-TGTCAAACTCACTCATGGCTTT	56	310
*TNF*-*α*	NM_001003244	FW-TGACAAGCCAGTAGCTCATGTT RV-CGGCAAAGTCCAGATAGTTAGG	56	410
*IL*-*2*	NM_001003305	FW-TTGTCGCAAACAGTGCACCTA RV-CCTGGAGAGTTGGGGGTTCT	60	131
*IL*-*4*	NM_001003159	FW- CTCACCAGCACCTTTGTCCA RV- GTCAGCTCCATGCACGAGTC	60	107
*IFN*-*β*	NM_001135787	FW- CAGTAGATGCATCCTCCAAACA RV- GACTATTGTCCAGGCACAGATG	55.9	493
*IFN*-*γ*	NM_001003174	FW-GCTGTAACTGTCAGGCCATGTTT RV-TGTTTTGTCACTCTCCTCTCTCCA	60	140

**Table 2:** **Primers sequences used for quantitative real time PCR (qRT-PCR)**

Gene	Accession number	Sequence (5′–3′)	Amplicon size (bp)
*NFKB*	NM_001003344.1	FW-GGAGGAGACCGGCAGCTTA RV-GCCGGTGCTATCTGGAAGAA	129
*IRF3*	XM_005616307.1	FW-TCACCACGCTACACCCTCTG RV-ATTTCCAGCAGGGCCCTAAG	119
*IRF7*	XM_005631711.1	FW-GTCGGGGCTCCCCATACTAC RV-CCCTTCTCGCTGCACGTACT	143
*TNF*-*α*	NM_001003244.4	FW-CTGCCTCAGCCTCTTCTCCTT RV-CTGGGCAAGAGGGCTGATTA	133
*IL*-*1β*	NM_001037971	FW-GAAGAAGCCCTGCCCACA RV-AATTATCCGCATCTGTTTTGCAG	104
*IL*-*1α*	NM_001003157.2	FW-TTGTGAGTGCCCAAAATGAAGA RV-CCATGACGCTCCCAAAAGA	109
*IL*-*6*	NM_001003301	FW-CTGGCAGGAGATTCCAAGGAT RV-TCTGCCAGTGCCTCTTTGC	167
*IL*-*8*	NM_001003200	FW-TTGCTCTCTTGGCAGCTTTTG RV-TTTGGGATGGAAAGGTGTGG	122
*IL*-*10*	NM_001003077	FW-ACATCAAGAACCACGTGAACTCC RV-ACTCACTCATGGCTTTGTAGACACC	177
*TGF*-*β1*	NM_001003309	FW-AGCCCGAGGCGGACTACTAC RV-CGGAGCTCTGATGTGTTGAAGA	130
*IFN*-*β*	NM_001135787.1	FW-GAAGCTCCACTGGCAGAAGG RW-TGGCCTTCAGGTACTGCACA	137
*CXCL10*	NM_001010949.1	FW-TCCTGCAAGTCCATCGTGTC RV-ATTGCTTTCACTAAACTCTTGATGGTC	114
*RPL27*	NM_001003102	FW-TCGTCAACAAGGATGTCTTCAGAG RV-TCTTGCCAGTCTTGTACCTCTCCT	96

Real-time PCR was performed in duplicate wells on StepOnePlus™ (Applied Biosystems), using the universal temperature cycles as suggested by the manufacturer. Melting curves were acquired to ensure that a single product was amplified in the reaction. All PCR reactions were carried out in 96-well optical reaction plates (Applied Biosystems, Warrington, UK) with 1× Power SYBR^®^ Green PCR Master Mix (Applied Biosystems), 1 ng (endometrial tissue) or 2 ng (cell cultures) of cDNA, 80 nM of each primer in a total reaction volume of 12.5 μL. After analyzing the melting curves, the PCR products were run through a 2.5% gel agarose to confirm the expected product size. The identity of PCR products was initially confirmed by DNA sequencing. The data of relative mRNA quantification was analyzed with the real-time PCR miner algorithm [17] and with 2^−ΔΔ*C*T^ method (for *IL*-*10*, *INFβ*, *IL*-*1α*) [18]. Results of qRT-PCR are expressed as the fold increase relative to the unstimulated control.

#### Immunofluorescence

After the stimulation assay, epithelial and stromal cells were washed three times in sterile PBS, fixed for 10 min in 4% paraformaldehyde (Sigma), washed two times in PBS- 0.1% Triton for 5 min and incubated with the blocking solution (PBS + 2.5% BSA) for 1 h at room temperature. Cells were incubated overnight at 4 °C with rabbit polyclonal anti-IRF3 antibody (1:50, ab25950, Abcam^®^, Cambridge, UK) and rabbit polyclonal anti-NFkB p65 antibody (1:100, sc-109-G, Santa Cruz biotechnology^®^, Texas, USA). After washing twice for 10 min in PBS, the secondary antibody (1:300, Alex Fluor^®^ 594 Goat Anti-Rabbit IgG (H + L), Life Technologies, Carlsbad, USA) was added and cells incubated for 1 h at room temperature. After washing for 10 min in PBS, the glass coverslips were mounted in Vectashield^®^ mounting medium with DAPI (H-1200, Vector Laboratories Inc., Burlingame, CA, USA). Slides were observed using a fluorescence microscope (Leica DM5000B) and the images obtained in Adobe Photoshop CS5.

### Statistical analysis

Data were analyzed through a statistical software package (Statistica 5.0, StatSoft Inc., Tulsa, OK, USA, 1995). Categorical data was analyzed by Fisher’s exact test. Hemogram, blood biochemical, cell number and cytokine transcription data were log-transformed (log x + 1) to normalize distribution. Hemogram, blood biochemical and endometrial cytokine transcription data (Experiment 1) and cell number (Experiment 2) were analyzed by ANOVA. In stromal cells Pyo18 induced cell death after 4 h of incubation, so transcription data was not available. Therefore, two different datasets were considered in Experiment 2. One dataset excluded data from stimulation with Pyo18. This dataset was analyzed using the MANOVA factorial procedures with three fixed effects: type of cells (*n* = 2; stromal cells, epithelial cells); type of stimulus (*n* = 3; LPS, Pyo14, Pyo18Δ*hlyA*); time of incubation (1, 4 h) and their interactions. Additionally, a second dataset excluded data from the 4-h endpoint. This dataset was analyzed by a MANOVA factorial procedure with two fixed effects: type of cells (*n* = 2; stromal cells, epithelial cells) and type of stimulus (*n* = 4; LPS, Pyo14, Pyo18Δ*hlyA*, Pyo18). Significant effects were further analyzed and means compared using the Scheffé test. Significance was determined at the 5% confidence level (*p* < 0.05).

## Results

### Hemolytic *E. coli* induces a more extensive uterine damage and inflammatory cell infiltration than non-hemolytic *E. coli* (Experiment 1)

**Figure 1:**
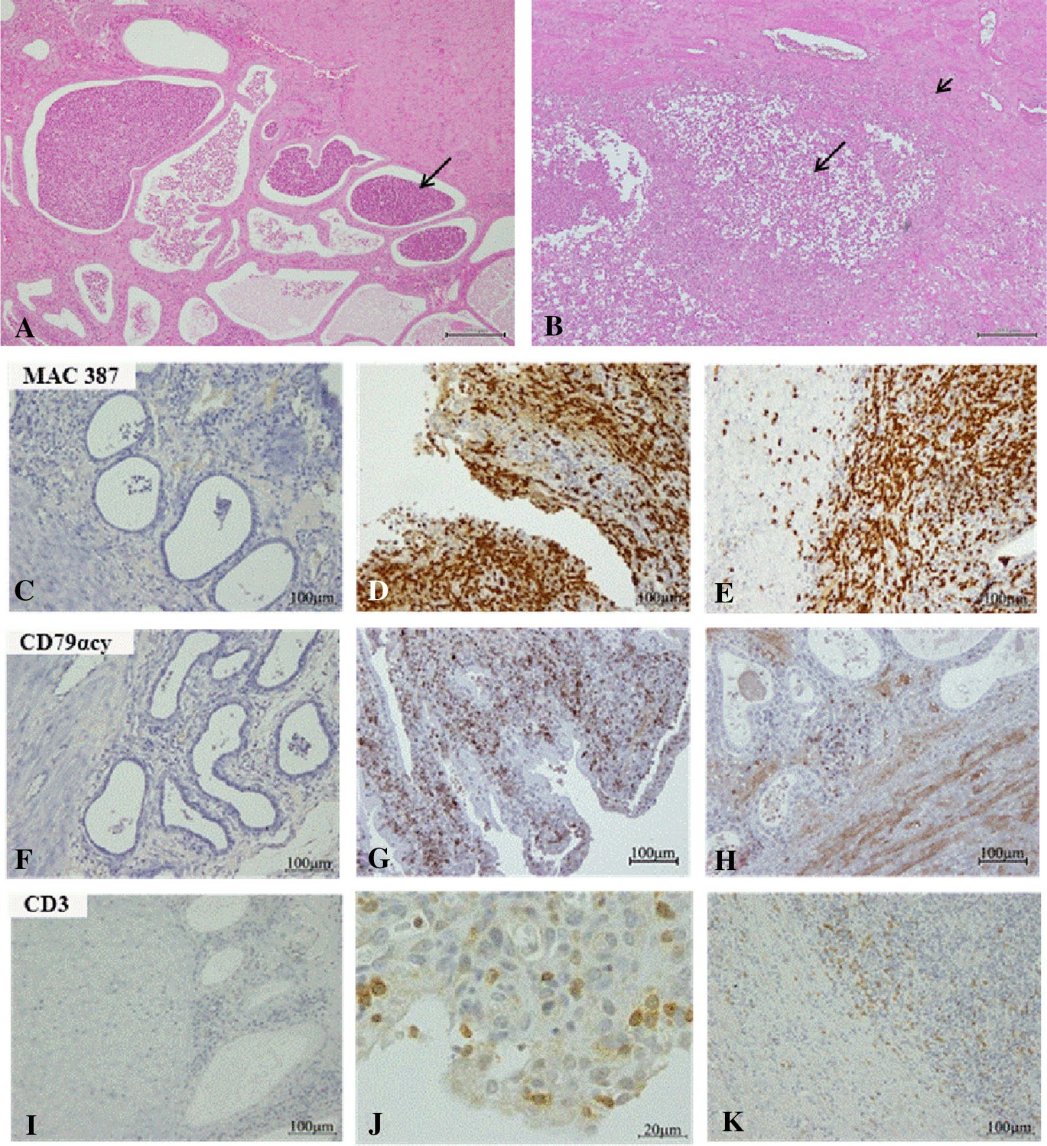
**Histology of uteri from bitches diagnosis with pyometra.** **A**–**B** Histological section of uterine pyometra samples. **A** non-hemolytic *E. coli* pyometra evidencing basal glands with myeloid cells infiltration (arrow); **B** β-hemolytic *E. coli* pyometra evidencing extensive damage of basal glands (arrow) and metritis (arrow head). Staining by H&E. **C**–**K** Immunostaining of calprotectin positive cells (myeloid cells), CD79 αcy positive cells (B lymphocytes) and CD3 positive cells (T lymphocytes) in pyometra uterine sections. Myeloid cells (granulocytes/macrophages) detection: **C** negative control (staining with mouse isotype); **D**, **E** infiltration in apical and basal layer, respectively (cytoplasmic staining pattern). B lymphocytes detection: **F** negative control (PBS); **G**, **H** infiltration in apical and basal layer, respectively (membrane staining pattern). T lymphocytes detection: **I** negative control (staining with rabbit isotype); **J**, **K** infiltration in apical and basal layer (membrane and cytoplasmic staining pattern), respectively.

Hemolytic and non-hemolytic *E. coli* pyometra bitches showed similar hematological and biochemical blood results (Table 3). All pyometra uterine samples showed destroyed luminal epithelium, damaged apical glands, inflammatory cellular infiltrate in the stromal and glandular compartments and cystic endometrial glands. Interstitial edema, blood extravasation and destroyed basal glands were observed in 52–68% of cases. However, 6 of 8 (75%) of hemolytic *E. coli* pyometra samples had histological evidence of metritis, whereas this was only observed in 2 of 10 (20%) of non-hemolytic *E. coli* pyometra cases (*p* < 0.05) (Figures 1A and B).

**Table 3:** **Hematological and biochemical parameters in bitches with hemolytic** ***E. coli*** **pyometra (** ***n*** **=** **8) and with non-hemolytic** ***E. coli*** **pyometra (** ***n*** **=** **10)**

	Hemolytic *E. coli* pyometra (Mean ± SEM)	Non-hemolytic *E. coli* pyometra (Mean ± SEM)
Hematological parameters		
White blood cell count (10^9^ l^−1^)	45.1 ± 5.5	33.8 ± 9.1
Eritrocytes (×10^12^ l^−1^)	5.5 ± 0.1	6.1 ± 0.4
Platelet count (10^9^ l^−1^)	216.1 ± 66.3	247.4 ± 48.7
Hemoglobin (g l^−1^)	126.0 ± 2.8	137.4 ± 7.8
Hematocrit (%)	37.3 ± 0.9	40.2 ± 2.4
Mean corpuscular volume (fL)	67.6 ± 0.9	66.1 ± 1.0
Mean corpuscular hemoglobin (pg)	22.9 ± 0.4	22.6 ± 0.2
Mean corpuscular hemoglobin concentration (g/dl)	33.9 ± 0.6	34.2 ± 0.4
Neutrophils (10^9^ l^−1^)	34.8 ± 4.2	25.8 ± 7.3
Band Neutrophils (10^9^ l^−1^)	0.9 ± 0.7	2.2 ± 1.1
Lymphocytes (10^9^ l^−1^)	3.4 ± 0.5	2.3 ± 0.4
Monocytes (10^9^ l^−1^)	5.65 ± 1.8	3.2 ± 0.9
Eosinophils (10^9^ l^−1^)	0.2 ± 0.1	0.3 ± 0.2
Basophils (10^9^ l^−1^)	0.3 ± 0.3	0
Biochemical parameters		
Alanine-aminotransferase (U L^−1^ at 25 °C)	22.5 ± 3.9	38.1 ± 12.5
Alkaline phosphatase (U L^−1^)	309.2 ± 146.5	488.1 ± 311.8
Creatinine (μmol L^−1^)	114.9 ± 17.7	97.2 ± 8.8
Urea (mmol L^−1^)	5.1 ± 1.6	6.4 ± 2.9

For all parameters *p* > 0.05.

An extensive stromal infiltration of myeloid cells (granulocytes/macrophages) (Figures 1D and E) and B lymphocytes was observed, being B lymphocytes more predominant in the apical than in the basal layer (Figures 1G and H). Stromal infiltration of T lymphocytes was moderate and more predominant in the apical layer (Figures 1J and K). Most (84%) of the pyometra samples also showed an extensive infiltration of myeloid cells in endometrial glands, whereas the glandular infiltration of lymphocytes was absent or weak. The presence of inflammatory cells in normal diestrous uteri was scarce (detected in 30% of the samples) and consisted of few granulocytes/macrophages (apical and basal layer) and T cells (apical layer) in the stromal compartment.

### Hemolytic and non-hemolytic *E. coli* pyometra endometria have different transcription levels of genes coding pro-inflammatory cytokines (Experiment 1)

**Figure 2:**
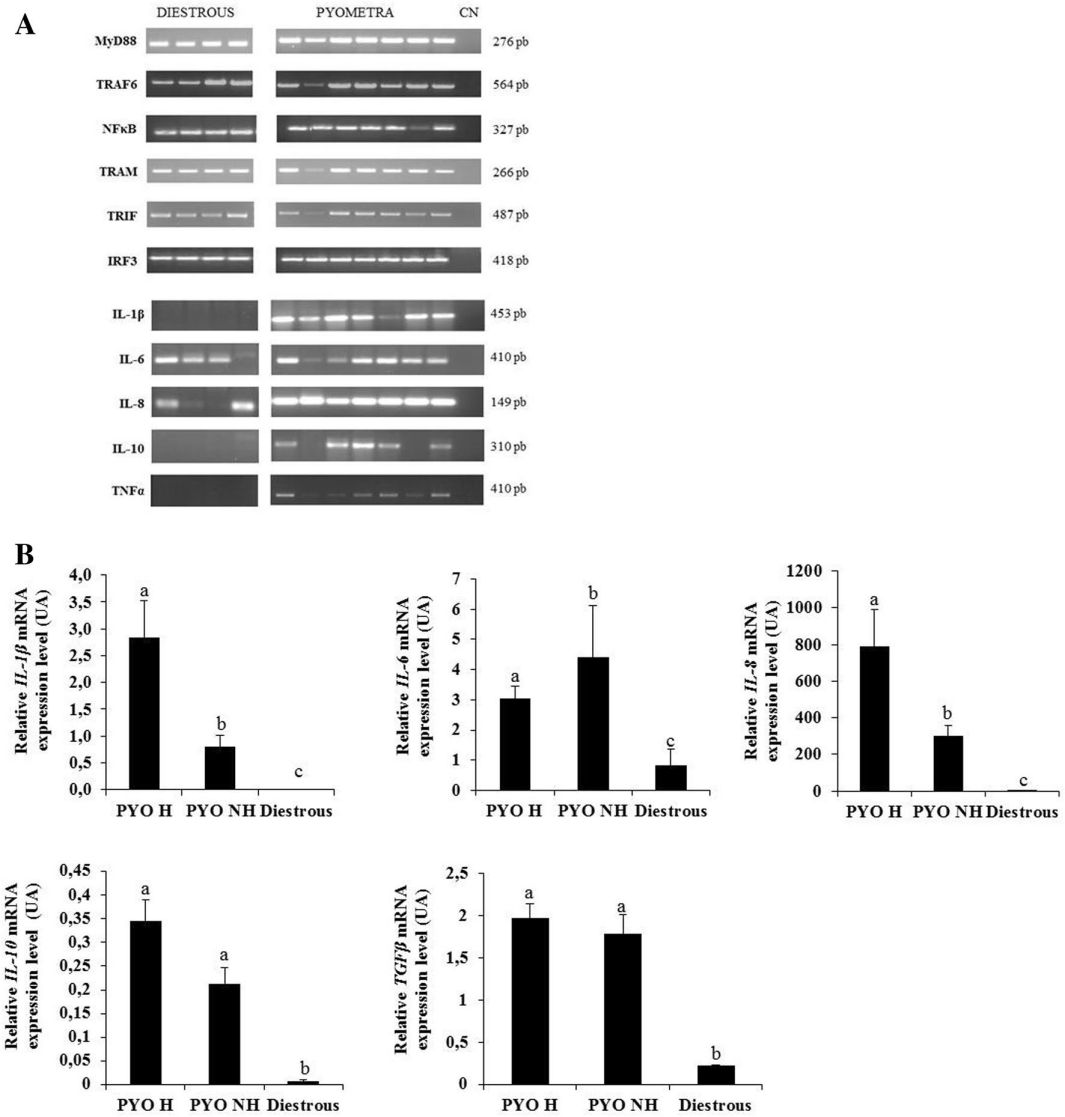
**Transcription of TLRs signaling components in diestrous and pyometra endometria. A** Representative PCR detection of transcripts of TLRs signaling components and of cytokines in diestrous and pyometra endometria. *NC* negative control. **B** Relative mRNA expression level (arbitrary units, AU) of *IL*-*1β*, *IL*-*6*, *IL*-*8*, *IL*-*10*, and *TGFβ* evaluated by real-time PCR, in hemolytic *E. coli* endometria, non-hemolytic *E. coli* endometria and diestrous endometria. Data are given as mean ± SEM. Columns with different superscripts differ significantly (*IL*-*1β*, *IL*-*6*, *IL*-*8* ^a vs b^ *p* < 0.01^a,b vs c^; *p* < 0.00001; *IL*-*10*, *TGFβ* ^a vs b^ *p* < 0.0001). MyD88 (myeloid differentiation factor 88); TRAM (TRIF related adaptor molecule); TRAF6 (TNFR-associated factor 6); TRIF (TIR domain-containing adaptor inducing interferon (IFN)-β); IRF3 (interferon regulatory factor 3).

Constitutive transcription of MyD88-dependent (*MyD88*, *TRAF6*, *NFKβ*) and independent (*TRAM*, *TRIF*, *IRF3*) pathway components was detected in healthy diestrous and pyometra endometria (Figure 2A). *IL*-*2*, *IL*-*4*, *IFNβ* and *IFNγ* transcripts were not detected in both endometria types. Therefore, *MyD88*, *TRAM*, *TRAF6*, *TRIF*, *NFκB*, *IRF3*, *IL*-*2*, *IL*-*4*, *IFNβ* and *IFNγ* genes were not analysed by qRT-PCR.

Overall, transcription levels of pro-inflammatory cytokines *IL*-*1β*, *IL*-*6* and *IL*-*8*, and of anti-inflammatory cytokines *IL*-*10* and *TGFβ* were significantly higher (*p* < 0.0001) in pyometra endometria than in healthy diestrous endometria. However, hemolytic *E. coli* pyometra endometria had higher transcription levels of *IL*-*1β* and *IL*-*8* and lower transcription levels of *IL*-*6* than non-hemolytic *E. coli* endometria (*p* < 0.01) (Figure 2B).

### Cytotoxicity due to α-hemolysin is mainly targeted to stromal endometrial cells (Experiment 2)

In epithelial cells, bacterial adhesion after 1 and 4 h of incubation was similar (3-5%) for both *E. coli* strains. This low bacterial adhesion was also observed in stromal cells incubated with Pyo14. In both epithelial and stromal cells incubated with Pyo14, bacterial internalization was absent (at 1 h) or very low (at 4 h; 0.1 – 0.2%). Incubation of stromal cells with Pyo18 for 4 h resulted in the death of most cells, therefore bacterial adhesion and internalization could not be evaluated.

**Figure 3:**
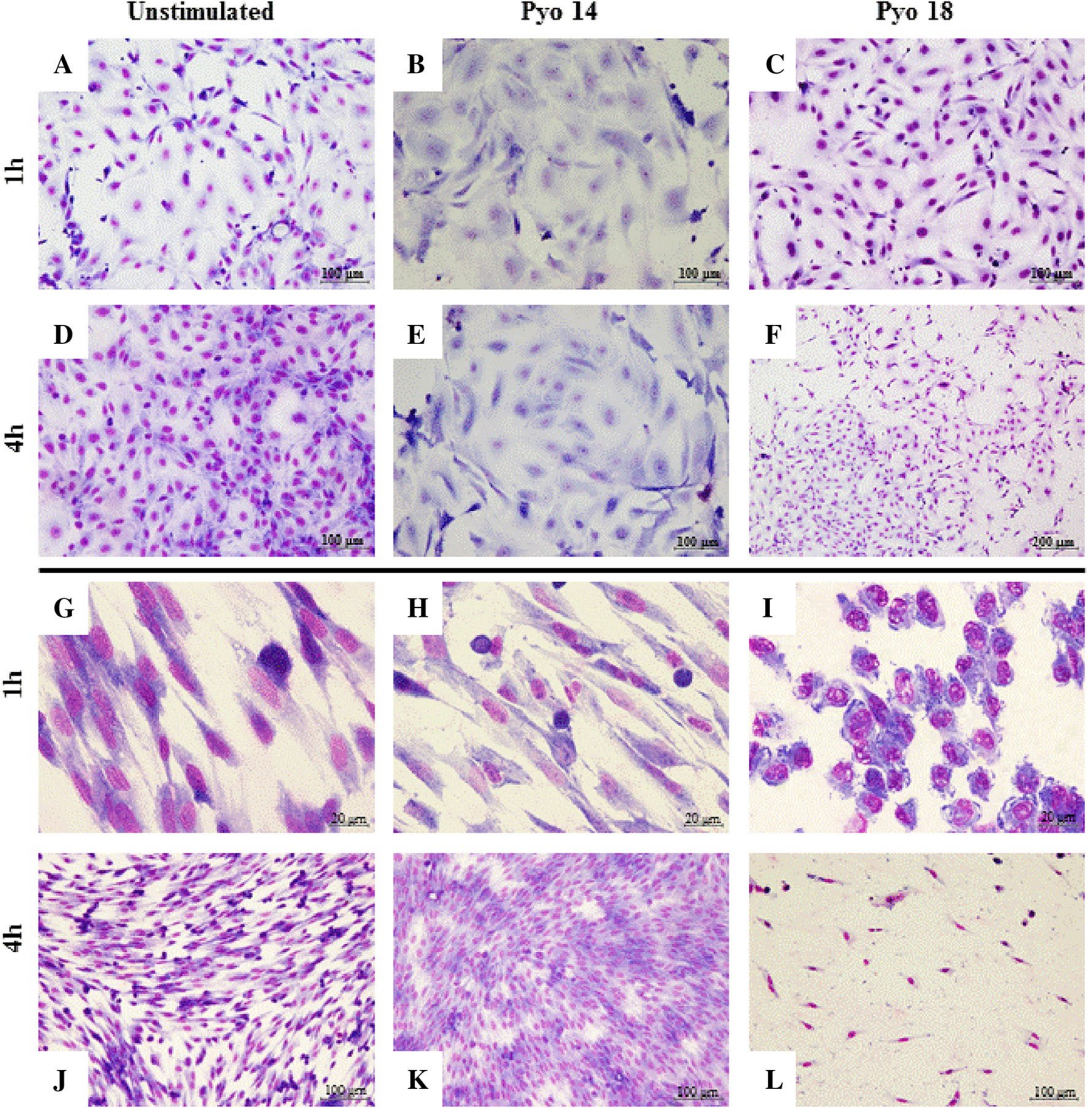
**Effect of hemolytic** ***E. coli*** **in epithelial and stromal cells.** Morphology of endometrial epithelial (**A**–**F**) and stromal (**G**–**L**) cell cultures stained with Giemsa after 1 and 4 h of incubation: unstimulated cells (**A**, **D**, **G**, **J**); cells incubated with *Pyo14* (**B**, **E**, **H**, **K**) and *Pyo18* (**C**, **F**, **I**, **L**).

**Figure 4:**
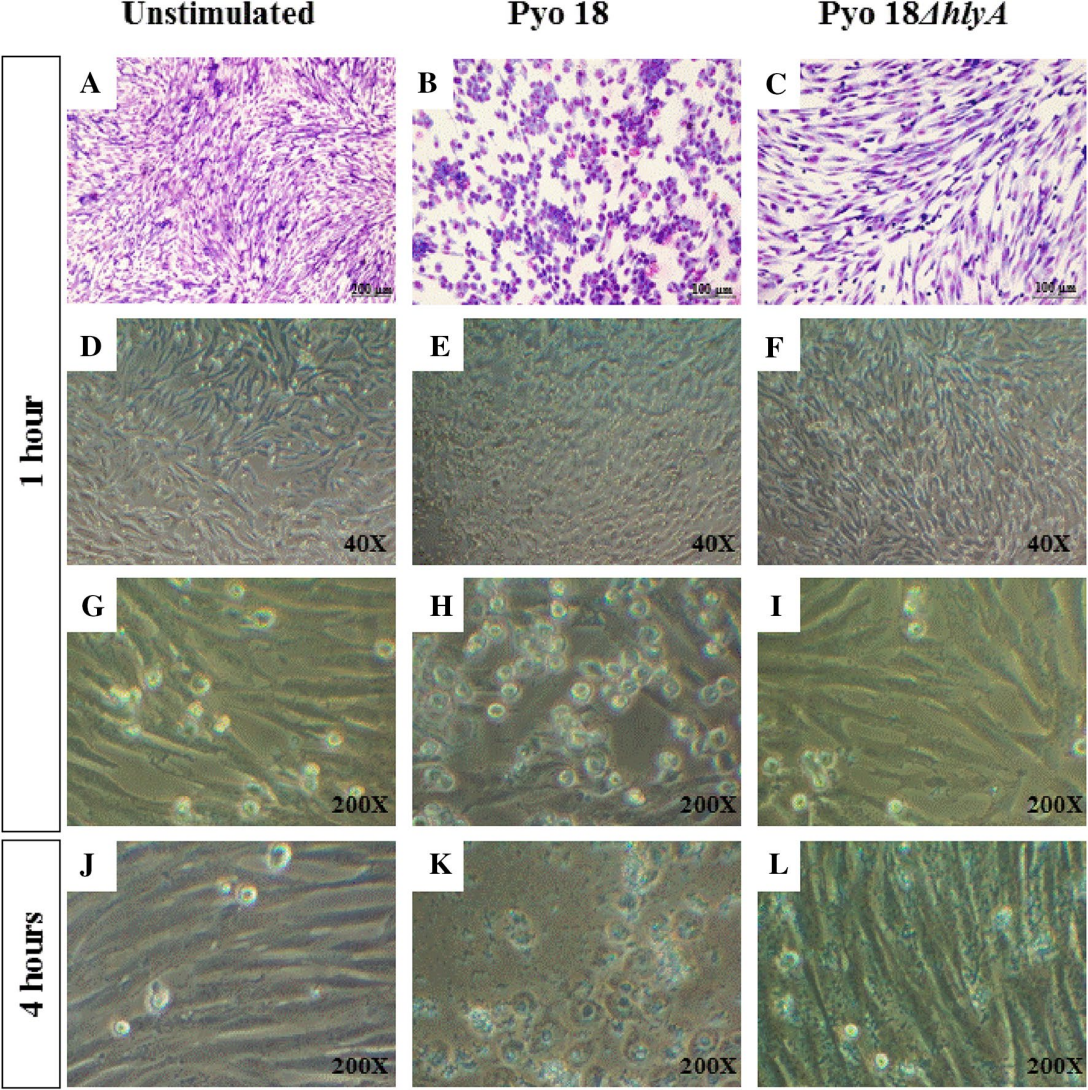
**Effect of Pyo18 isogenic** ***hlyA*** **deletion mutant (Pyo18ΔhlyA) in epithelial and stromal cells.** Morphology of endometrial stromal cells after 1 and 4 h of incubation, evaluated after Giemsa staining (**A**–**C**) or under phase-contrast (**D**–**L**): unstimulated cells (**A**, **D**, **G**, **J**); cells incubated with *Pyo18* (**B**, **E**, **H**, **K**); cells incubated with *Pyo18ΔhlyA* (**C**, **F**, **I**, **L**).

Incubation with Pyo14 did not affect cell numbers and cell morphology in both types of cells (Table 4; Figures 3B, E, H, K). Incubation with Pyo18 induced a decrease in epithelial cell numbers at 4 h (*p* < 0.0001; Table 4), although the morphology of surviving cells was not affected (Figures 3C and F). Incubation of stromal cells with Pyo18 for 1 h did not affect cell numbers (Table 4), but cells lost the typical elongated fibroblast shape, becoming spherical (Figures 3I, 4B, E, H). As stated above, incubation of stromal cells for 4 h with Pyo18 induced the detachment and death of all the cells (Figures 3L and 4K).

**Table 4:** **Effect of bacterial or LPS incubation on endometrial epithelial and stromal cell numbers**

*E. coli* strain	Stromal cells		Epithelial cells	
	1 h (%)	4 h (%)	1 h (%)	4 h (%)
Pyo14	102.7 ± 5.9	98.9 ± 2.5^*^	107.0 ± 1.4	96.3 ± 6.3^*^
Pyo18	102.9 ± 4.8^a^	0^b,**^	105.9 ± 3.3^c^	54.3 ± 3.4^d,**^
Pyo18Δ*hlyA*	104.5 ± 3.0	97.4 ± 3.2^*^	110.1 ± 2.3	97.9 ± 4.4^*^
LPS	100.7 ± 8.4	96.0 ± 4.6^*^	115.8 ± 2.2	99.9 ± 6.4^*^

Statistical differences were analyzed by ANOVA. ^a vs b^ *p* < 0.00001 for stromal cells. ^c vs d^ *p* < 0.0001 for epithelial cells. Within columns, different superscripts show significant differences: ^* vs **^ *p* < 0.01. Results of cell counts for each type of stimulation are presented as the mean percentage relative to the respective unstimulated control (*n* = 3 uteri with two replicates for all stimuli).

In contrast, incubation with the isogenic mutant Pyo18Δ*hlyA* did not induce a cytotoxic effect on stromal cells (Figures 4C, F, I, L). In fact, following incubation with this mutant strain, cell morphology and numbers were similar to those observed after incubation with Pyo14 (Table 4; Figures 3 and 4).

### Hemolytic and non-hemolytic *E. coli* strains induce differential immune response in endometrial epithelial and stromal cells

**Figure 5:**
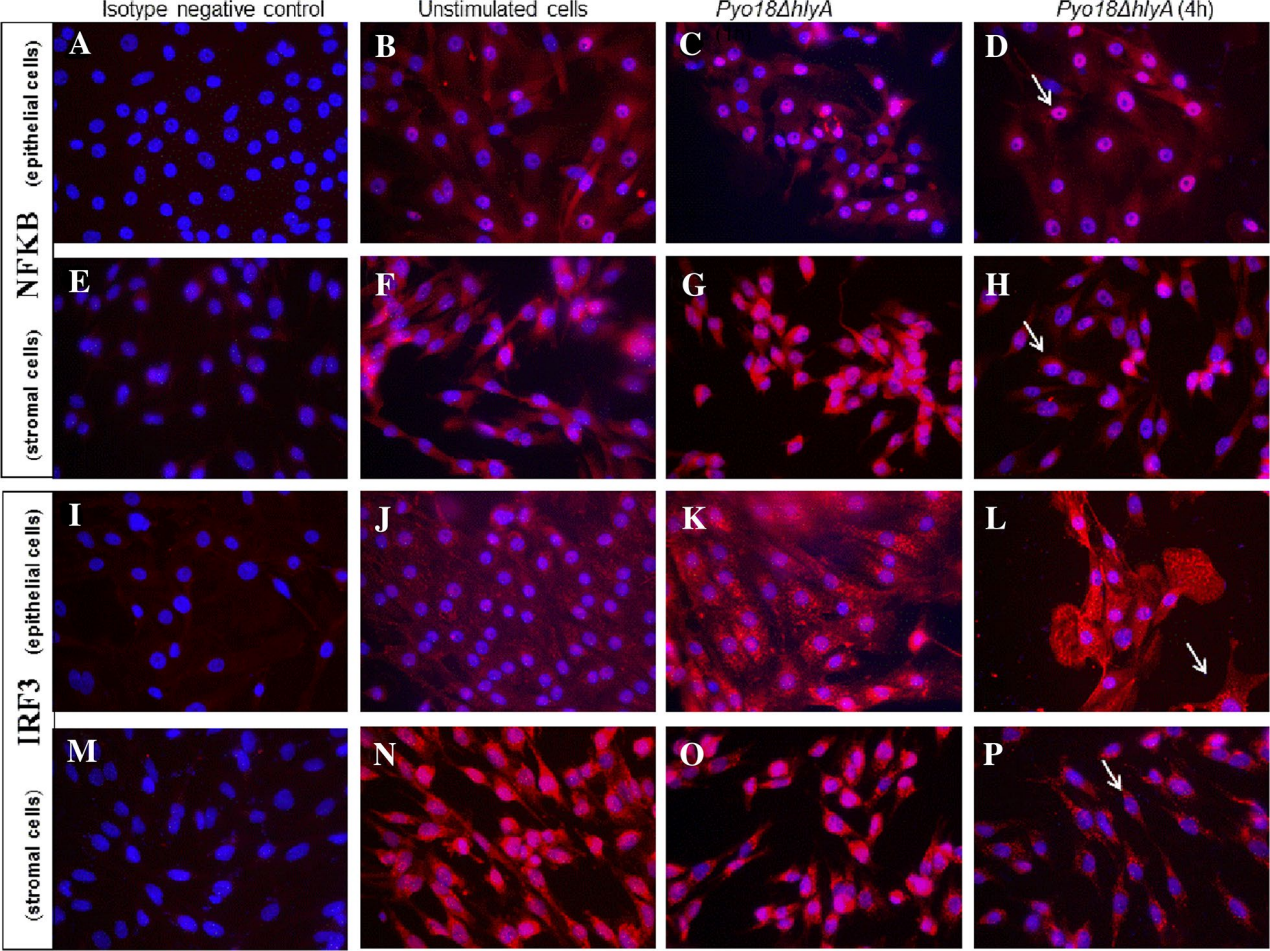
**Activation of TLRs signaling pathways evaluated by immunofluorescence.** Nuclear detection of NFκB (**A**–**H**) and IRF3 (**I**–**P**) in endometrial epithelial and stromal cells by immunofluorescence. **A**–**D** and **I**–**L** epithelial cells [**A**, **I** rabbit isotype negative control after 4 h of incubation; **B**, **J** unstimulated cells after 4 h of incubation; **C**, **D** and **K**, **L** stimulation with *Pyo18ΔhlyA* strain during 1 h (**C**, **K**) and 4 h (**D**, **L**)]; **E**–**H** and **M**–**P** stromal cells [**E**, **M** rabbit isotype negative control after 1 h of incubation; **F**, **N** unstimulated cells after 1 h of incubation; **G**, **H** and **O**, **P** stimulation with *Pyo18ΔhlyA* strain during 1 h (**G**, **O**) and 4 h (**H**, **P**)]. White arrow—nuclear staining.

Transcription of *IRF3*, *IRF7* and *NFkB* genes was not affected by type of cells, type of bacteria or incubation time (data not shown). Translocation of IRF3 and NFκB to the nucleus was detected by IHC (Figure 5) in endometrial epithelial and stromal cells.

To allow depiction of the absolute transcription of cytokine genes in endometrial and stromal cells, Table 5 shows the Cycle threshold (Ct) values of unstimulated cells (negative controls) after 1 h and 4 h of incubation.

**Table 5:** **Mean cycle threshold (Ct) values of cytokine gene transcription in unstimulated endometrial epithelial and stromal cells following 1 and 4** **h of incubation**

	Epithelial cells		Stromal cells	
	1 h	4 h	1 h	4 h
Cytokine				
* IL*-*1β*	33.22	33.91	34.75	34.56
* IL*-*1α*	29.39	29.67	29.99	30.00
* IL*-*6*	31.48	31.27	33.08	32.84
* TNFα*	32.28	32.35	35.52	35.11
* IL*-*8*	24.23	24.53	26.63	27.21
* CXCL10*	30.84	31.29	31.43	31.64
* IL*-*10*	33.66	33.98	32.56	32.52
* INFβ*	35.15	32.87	32.00	34.21
Housekeeping				
*RPL27*	20.84	20.95	20.29	20.37

*n* = 3 uteri and two replicates per uteri. The mean Ct value of the housekeeping gene *RPL27* was 20.62 ± 0.61 (Mean ± SD).

**Figure 6:**
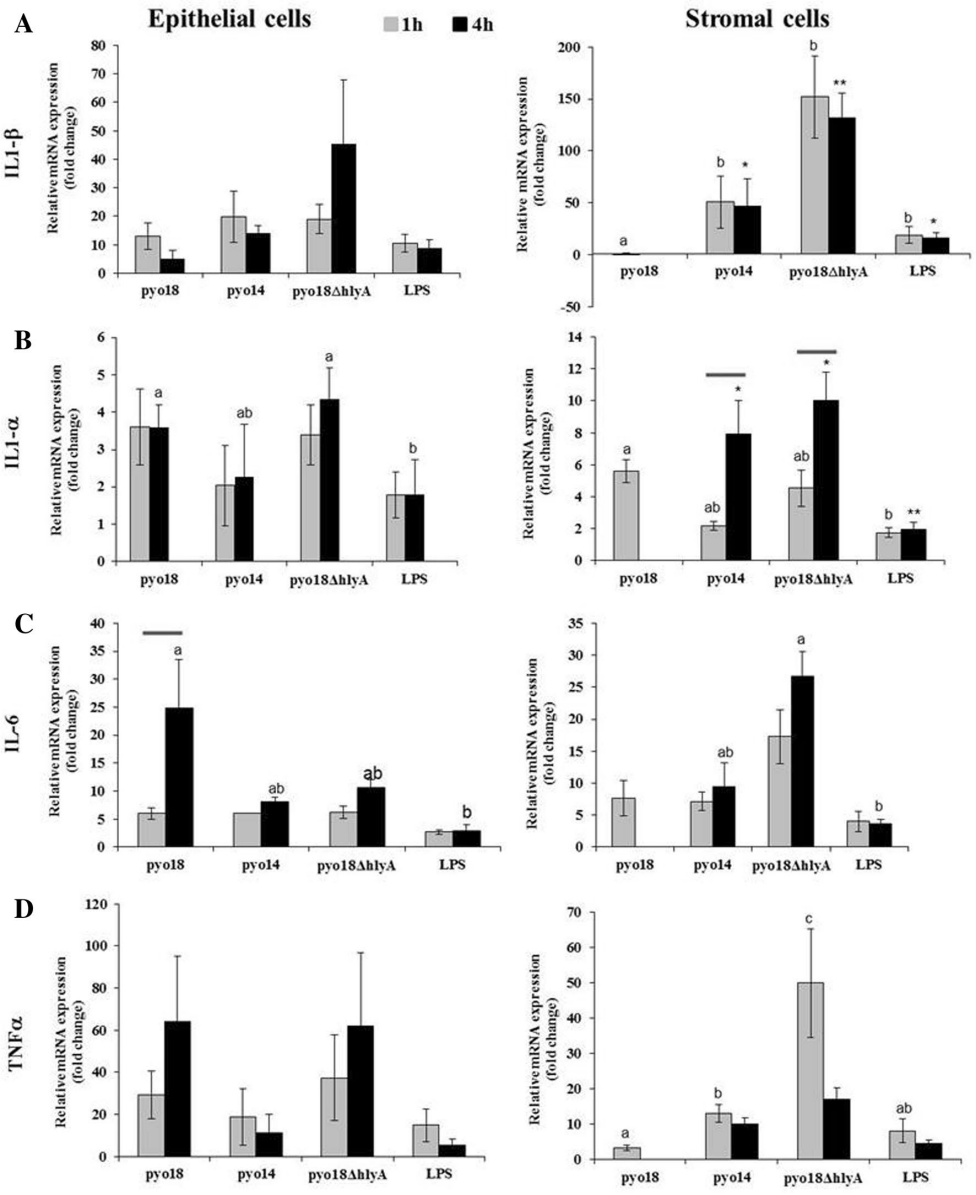
**Pro-inflammatory interleukins genes transcription in cultured canine endometrial epithelial and stromal cells after** ***Escherichia coli*** **or LPS cell stimulation.** Relative mRNA expression (qRT-PCR) of *IL*-*1β* (**A**), *IL*-*1α* (**B**), *IL*-*6* (**C**), *TNFα* (**D**) in cultured canine endometrial epithelial and stromal cells in response to hemolytic *E. coli* (Pyo18), non-hemolytic *E. coli* (Pyo14) and the isogenic mutant of Pyo18 (*Pyo18ΔhlyA*). Treatment with 1 μg/mL of LPS was used as a positive control. Data are mean ± SEM (*n* = 3 uteri with two replicates for all stimuli). Transcription levels (fold increase) are normalized with those of non-stimulated cells ^a vs b^ *p* < 0.05 [(Epithelia cells: *IL*-*1α*, *IL*-*6*; stromal cells: *IL*-*1β*, *IL*-*1α*, *TNFα*]; ^a vs b^ *p* < 0.01 (stromal cells: *IL*-*6*); ^a vs c^ *p* < 0.05 (stromal cells: *TNFα*); * ^vs^ **p < 0.05 (*IL*-*1β*) * ^vs^ **p < 0.01 (*IL*-*1α*); *p* < 0.05 (1 vs 4 h: *IL*-*1α*, stromal cells); *p* < 0.01 (1 vs 4 h: *IL*-*6*, epithelia cells).

**Figure 7:**
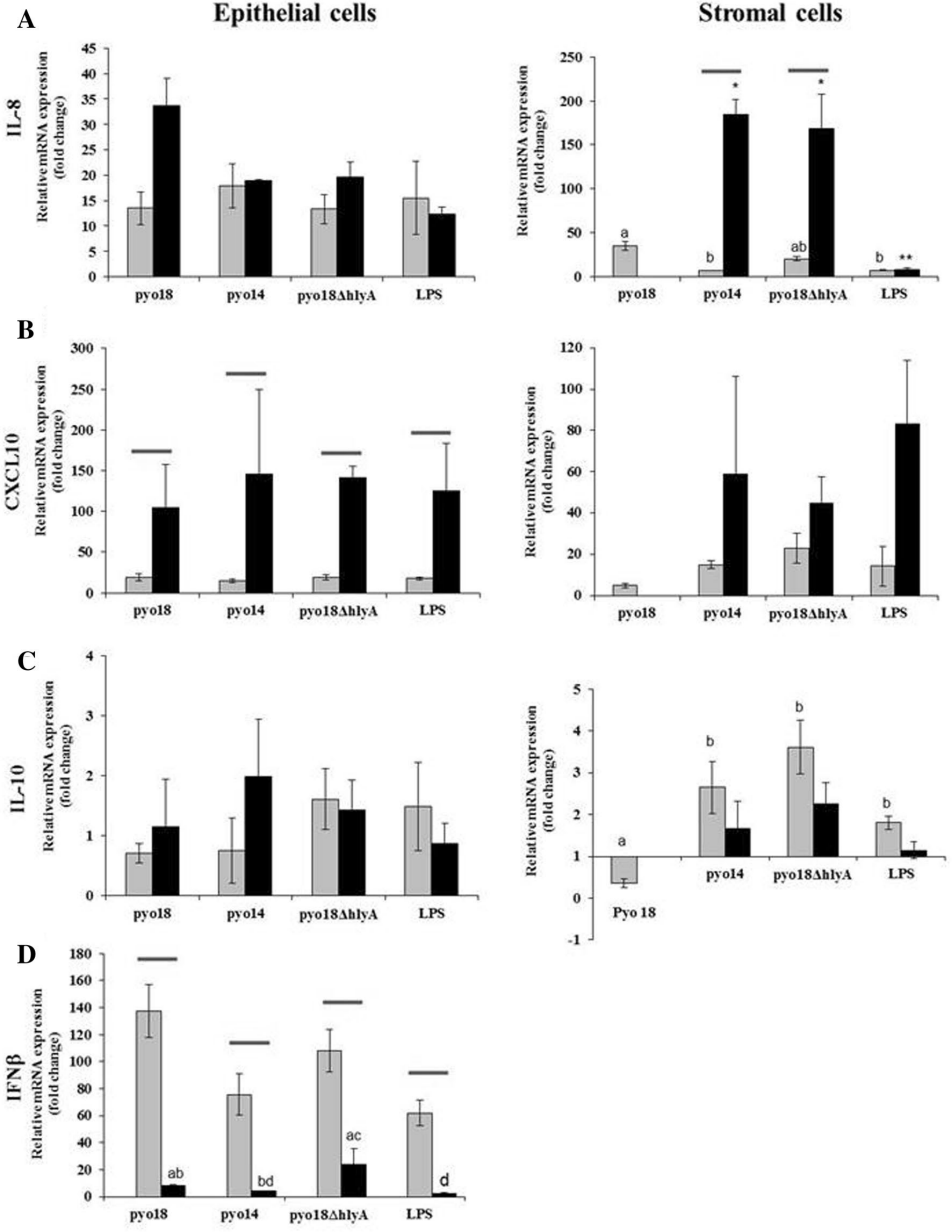
**Chemokines, anti-inflammatory Interleukin-10 and Interferon-β genes transcription in epithelial and stromal cells after** ***Escherichia coli*** **or LPS cell stimulation.** Relative mRNA expression (qRT-PCR) of *IL*-*8* (**A**), *CXCL10* (**B**), *IL*-*10* (**C**), *INFβ* (**D**) in cultured canine endometrial epithelial and stromal cells in response to hemolytic *E. coli* (Pyo18), non-hemolytic *E. coli* (Pyo14) and the isogenic mutant of Pyo18 (*Pyo18ΔhlyA*). Treatment with 1 μg/mL of LPS was used as a positive control. Data are mean ± SEM (*n* = 3 uteri with two replicates for all stimuli). Transcription levels (fold increase) are normalized with those of non-stimulated cells. ^a vs b^ *p* < 0.05 (stromal cells: *IL-8*); ^a vs d^ *p* < 0.05 (epithelial cells: *IFNβ*); ^c vs d^ *p* < 0.05 (Epithelial cells: *IFNβ*); ^b vs c^ *p* < 0.05 (epithelial cells: *IFNβ*); ^a vs b^ *p* < 0.01 (stromal cells: *IL-10*); * ^vs^ **p < 0.01 (stromal cells: *IL-8*); *p* < 0.01 (1 vs 4 h: *IL-8*, stromal cells; *CXCL10*, epithelial cells); *p* < 0.001 (1 vs 4 h: *IFNβ*, epithelial cells).

In epithelial cells, Pyo18, Pyo18*ΔhlyA* and Pyo14 induced similar transcription levels of cytokines in each endpoint of incubation (Figures 6 and 7). Stimulation with Pyo18, induced a higher *IL*-*6* (1 h vs 4 h: 6.0 vs 24.9 fold increase, *p* < 0.01) (Figure 6C) mRNA expression after 4 h than after 1 h of incubation. All bacterial stimuli and LPS induced a significant increase in transcription levels of *CXCL10* after 4 h (1 vs 4 h: Pyo18–19.2 vs 105.2; Pyo14–14.9 vs 146.5; Pyo18*ΔhlyA*—19.3 vs 142.4; LPS—17.8 vs 126.3 fold increase, *p* < 0.01) (Figure 7B), and a decrease of *IFNβ* after 4 h (1 vs 4 h: Pyo18—137.4 vs 7.8; Pyo14—75.5 vs 4.0; Pyo18*ΔhlyA*—108.2 vs 24.1; LPS 62.0 vs 2.5 fold increase, *p* < 0.001) (Figure 7D).

In stromal cells, after 1 h of incubation and compared to the isogenic mutant Pyo18Δ*hlyA* and to Pyo14, respectively, Pyo18 induced a lower transcription level of *IL*-*1β* (0.99 vs 152.0 vs 50.9 fold increase, *p* < 0.001) (Figure 6A), *TNFα* (3.2 vs 49.9 vs 12.9 fold increases, *p* < 0.05) (Figure 6D) and *IL*-*10* (0.4 vs 3.6 vs 2.6 fold increases, *p* < 0.001) (Figure 7C). Stimulation with Pyo14, induced higher *IL*-*1α* and *IL*-*8* transcription levels after 4 h than 1 h of incubation (1 h—2.2 vs 4 h—7.9 fold increase, *p* < 0.05 and 1 h—19.8 vs 4 h—184.1 fold increase, *p* < 0.01, respectively) (Figures 6B and 7A). Stimulation with Pyo18Δ*hlyA* also induced higher *IL*-*1α* and *IL*-*8* transcription levels after 4 h than 1 h of incubation (1 h—4.5 vs. 4 h—9.9 fold increase, *p* < 0.05 and 1 h—4.5 vs. 4 h—168.7 fold increase, *p* < 0.01, respectively) (Figures 6B and 7A). Transcription of *INFβ* was not detected in stromal cells.

The bacterial stimuli induced different gene transcription levels in endometrial epithelial and stromal cells. Pyo14 and Pyo18*ΔhlyA* induced higher transcription levels of *CXCL10* after 4 h of incubation in epithelial than in stromal cells (Pyo14—146.5 vs 58.9 fold increase, *p* < 0.05 and Pyo18*Δhly*—142.4 vs 44.9 fold increase, *p* < 0.01, respectively) (Figure 7B). Also, Pyo14 and Pyo18*ΔhlyA* induced higher transcription levels of *IL*-*8* in stromal than epithelial cells after 4 h of incubation (Pyo14—184.7 vs 6.7 fold increase; Pyo18Δ*hlyA*—168.7 vs 19.8 fold increase, respectively, *p* < 0.02) (Figure 7A).

## Discussion

The results of this study indicate that HlyA contributes to the virulence of *E. coli*, by inducing tissue damage and a compromised early uterine immune response. On one side, the cytotoxic activity towards epithelial and stromal cells may facilitate progression of bacteria into the uterine tissue. On the other side, the modulation of the uterine innate immune response may induce a delayed neutrophil influx to the uterus. Additionally, the known cytotoxic effect of HlyA in granulocytes and lymphocytes, as well as the enhancement of access to cell nutrients and iron storages [19], might also enhance hemolytic *E. coli* survival within the uterine tissue.

The relationship between the isolation of β-hemolytic *E. coli* and clinical signs and histopathological findings was not reported. UPEC strains that express HlyA were shown to cause more extensive tissue damage within the urinary tract, which was correlated with more severe clinical outcomes [20]. In this study, clinical signs, hematological and blood biochemical results were similar in hemolytic and non-hemolytic *E. coli* pyometra bitches. However, β-hemolytic *E. coli* was associated with high endometrial damage and metritis.

All *E. coli* pyometra uteri evidenced an infiltrate of inflammatory cells, including neutrophils, lymphocytes and macrophages. This may contribute for the up-regulation of TLR2 and TLR4 transcription [9, 10] and expression [8] and the consequent up-regulation of gene transcription of several pro- and anti-inflammatory cytokines, including *IL*-*1β*, *IL*-*6*, *IL*-*8*, *IL*-*10* and *TGFβ*. However, hemolytic *E. coli* endometria had higher transcription levels of *IL*-*1β* and *IL*-*8* and lower transcription levels of *IL*-*6* than non-hemolytic *E. coli* endometria. IL-6 stimulates leukocyte activation and myeloid progenitor cell proliferation, regulates the synthesis of acute phase proteins and is a powerful pyrogen [21]. In pyometra cases, C-reactive protein, serum amyloid A and haptoglobin concentrations can be used to monitor early post-OHE complications and ongoing inflammation [22]. C-reactive protein was also associated with SIRS [23]. However, in the above studies the hemolytic nature of *E. coli* was not evaluated. High serum levels of IL-8 were observed in pyometra bitches, especially in those that developed SIRS, suggesting IL-8 as a useful early biomarker of uterine infection and, possibly, of sepsis [3]. IL-8 is also a potent neutrophil chemotactic molecule. Neutrophil recruitment to the site of infection was shown to be critical for bacterial clearance in the uterus, although this may also lead to tissue damage [24].

Endometrial epithelial cells showed a high sensitivity to the cytotoxic effect of HlyA. This may lead to the suggestion that β-hemolytic *E. coli* induces an earlier damage to the epithelial layer than non-hemolytic *E. coli* strains. During uterine contamination, epithelial cell apoptosis [25] and/or degradation of proteins involved in cell–cell and cell–matrix interactions [26] due to the sublytic concentrations of HlyA may be the reason for the disruption of the epithelial layer. Additionally, the cytotoxic effect of HlyA may be associated with the observed destruction of epithelial glandular cells of pyometra uteri. Endometrial stromal cells showed a higher sensitivity to the cytotoxic effect of HlyA than epithelial cells. A higher sensitivity of bovine stromal cells to pyolysin-mediated cytolysis of *Trueperella pyogenes* was recently reported [27]. This sensitivity of both epithelial and stromal cells to the cytotoxic effect of HlyA may explain the observed significant association between β-hemolytic *E. coli* isolation and metritis.

Adhesion to epithelial and stromal cells was similar following bacterial incubation, irrespective of *E. coli* strain. The observed level of *E. coli* adhesion is similar to that reported for bovine endometrial cells [28]. Pyo14 and Pyo18 have genes for Type 1 fimbrae and S + F1C fimbriae, whereas the *papG* gene that codes for P fimbriated was only detected in Pyo18 [14]. Although adherence of *E. coli* to canine endometrial cells was shown to be mediated by Type 1 fimbriae [29], the targeted deletion of specific adhesin genes in a canine pyometra strain was compensated by the presence of other adhesins, which indicates functional redundancy among adhesins [30]. Bacterial internalization was absent or very low. This indicates that cell invasion is not necessary for inducing the cytotoxic effect of HlyA. Poor internalization of hemolytic *E. coli* was reported in HeLa and HTB-4 cell co-culture systems [31]. Low internalization was also observed in bovine endometrial epithelial and stromal cells following incubation with non-hemolytic *E. coli* during 4 h [28].

Canine endometrial epithelial and stromal cells express TLR4 [8] and TLR4 binding triggers a signaling cascade that culminates in the activation of NFkB and IRF3. The detection of NFkB and IRF3 in the nucleus, together with the observed cytokine transcription results, indicates that the TLR signaling pathway was activated.

Differential transcription of cytokine genes by endometrial stromal but not epithelial cells was observed following stimulation with hemolytic and non-hemolytic *E. coli* strains. In stromal cells, transcription levels of *TNFα*, *IL*-*1β* and *IL*-*10* were lower following stimulation with Pyo18 than following stimulation with Pyo18*ΔhlyA* and Pyo14. The decrease in *TNFα* is probably responsible for the observed low levels of *IL*-*1β.* König and König [32] reported a down-regulation of *TNFα* expression in human inflammatory cells stimulated with hemolytic *E. coli*. Recently, in a bacteremia mouse model, *HlyA* inhibited *IL-1β* secretion [33]. A functional impact of early *IL-10* in UPEC UTI mice model were suggested as *IL10* ^*−*^/^−^ mice had more UPEC and more severe bacteriuria [34]. Overall, the observed down-regulation in *TNFα, IL*-*1β* and *IL*-*10* transcription in stromal cells may be seen as an attempt of hemolytic *E. coli* to delay the activation of the immune response.

Differential cytokine gene transcription was also observed in endometrial epithelial and stromal cells. The transcription of *CXCL10* was significantly higher in endometrial epithelial than stromal cells. CXCL10 displays antimicrobial activity against *E. coli* [35], and likewise defensins, may act as the first line of defense in epithelia [36]. A high transcription level of *CXCL10* was observed in canine endometrial stromal cells after stimulation with *E. coli* and LPS [37]. In stromal cells, CXCL10 may be involved in the recruitment and potentiation of T-helper 1 response [38]. However, γδ^+^/CD8^−^ T lymphocytes were the only significant lymphocyte population detected in pyometra uteri [39].

Stromal cells had a higher transcription level of *IL*-*8* than epithelial cells after incubation for 4 h with Pyo14 and Pyo18*ΔhlyA*. A high transcription level of *IL*-*8* in canine stromal cells after *E. coli* and LPS stimulation was reported by Karlsson et al. [37]. This may indicate that, during uterine *E. coli* infection and after disruption of the epithelial layer of the endometrium, stromal cells are an important early source of *IL-8*, a major cytokine involved in the activation and recruitment of neutrophils.

Transcription of *INFβ* was not detected in endometria (Experiment 1), but was detected in endometrial epithelial cells (Experiment 2) after incubation with Pyo18, Pyo18*ΔhlyA*, Pyo14 and LPS. Transcription of *IFNβ* by endometrial epithelial cells after 1 h of bacterial stimuli is likely triggered by LPS and is probably responsible for the up-regulation of *CXCL10* transcription after 4 h of stimulation. The lack of transcription of *IFNγ* in normal diestrus and pyometra uteri is in accordance with the results of endometrial transcription and serum levels of *IFNγ* found in pyometra cases [3, 9].

In conclusion, β-hemolytic *E. coli* infection was associated with the occurrence of metritis and with higher uterine tissue damage than non-hemolytic *E. coli* infection. β-hemolytic *E. coli* pyometra endometria had higher gene transcription of *IL*-*1β* and *IL*-*8* and lower gene transcription of *IL*-*6* than non-hemolytic pyometra endometria. The cytotoxic effect of HlyA was more severe in endometrial stromal than in epithelial cells. Endometrial stromal cell damage by HlyA is a potential relevant step of *E. coli* virulence in the pathogenesis of pyometra. Additionally, the HlyA-induced decrease of cytokine transcription by stromal cells, potentially leading to a decrease of cytokine expression, which may occur during the early stages of infection, may allow bacteria to establish a niche prior to the activation of an adequate innate immune response.
